# Specific Microbiome Changes in a Mouse Model of Parenteral Nutrition Associated Liver Injury and Intestinal Inflammation

**DOI:** 10.1371/journal.pone.0110396

**Published:** 2014-10-20

**Authors:** J. Kirk Harris, Karim C. El Kasmi, Aimee L. Anderson, Michael W. Devereaux, Sophie A. Fillon, Charles E. Robertson, Brandie D. Wagner, Mark J. Stevens, Norman R. Pace, Ronald J. Sokol

**Affiliations:** 1 Department of Pediatrics, Section of Pulmonary Medicine, University of Colorado, Aurora, Colorado, United States of America; 2 Department of Pediatrics, Section of Gastroenterology, Hepatology and Nutrition, Digestive Health Institute, Children’s Hospital Colorado, University of Colorado School of Medicine, Aurora, Colorado, United States of America; 3 Department of Molecular, Cellular and Developmental Biology, University of Colorado, Boulder, Colorado, United States of America; 4 Department of Biostatistics and Informatics, Colorado School of Public Health, University of Colorado Denver, Aurora, Colorado, United States of America; Charité-University Medicine Berlin, Germany

## Abstract

**Background:**

Parenteral nutrition (PN) has been a life-saving treatment in infants intolerant of enteral feedings. However, PN is associated with liver injury (PN Associated Liver Injury: PNALI) in a significant number of PN-dependent infants. We have previously reported a novel PNALI mouse model in which PN infusion combined with intestinal injury results in liver injury. In this model, lipopolysaccharide activation of toll-like receptor 4 signaling, soy oil-derived plant sterols, and pro-inflammatory activation of Kupffer cells (KCs) played key roles. The objective of this study was to explore changes in the intestinal microbiome associated with PNALI.

**Methodology and Principal Findings:**

Microbiome analysis in the PNALI mouse identified specific alterations within colonic microbiota associated with PNALI and further association of these communities with the lipid composition of the PN solution. Intestinal inflammation or soy oil-based PN infusion alone (in the absence of enteral feeds) caused shifts within the gut microbiota. However, the combination resulted in accumulation of a specific taxon, *Erysipelotrichaceae* (23.8% vs. 1.7% in saline infused controls), in PNALI mice. Moreover, PNALI was markedly attenuated by enteral antibiotic treatment, which also was associated with significant reduction of *Erysipelotrichaceae* (0.6%) and a Gram-negative constituent, the S24-7 lineage of Bacteroidetes (53.5% in PNALI vs. 0.8%). Importantly, removal of soy oil based-lipid emulsion from the PN solution resulted in significant reduction of *Erysipelotrichaceae* as well as attenuation of PNALI. Finally, addition of soy-derived plant sterol (stigmasterol) to fish oil-based PN restored *Erysipelotrichaceae* abundance and PNALI.

**Conclusions:**

Soy oil-derived plant sterols and the associated specific bacterial groups in the colonic microbiota are associated with PNALI. Products from these bacteria may directly trigger activation of KCs and promote PNALI. Furthermore, the results indicate that lipid modification of PN solutions may alter specific intestinal bacterial species associated with PNALI, and thus suggest strategies for management of PNALI.

## Introduction

The human microbiota is a complex, highly personalized source of both benefit and disease. Many diseases with poorly understood pathogenesis and etiology potentially result from disturbances within the normal microbiota that result in enrichment of damaging organisms or the depletion of favorable ones (i.e. dysbiosis). Progress toward understanding mechanisms that result in dysbiosis has been impeded by the enormous complexity of the human microbiota both in health and disease, and traditional reliance on culture-based techniques. However, with the advent of high volume, culture-independent DNA sequence analysis of microbiota constituents, it becomes feasible to identify candidate causative microorganisms for previously unexplained diseases even in the complexity of the gut microbiota.

One cryptic disease that potentially has roots in perturbation of the intestinal microbiota results from use of parenteral nutrition (PN) [1] to treat infants with congenital or acquired intestinal diseases that limit tolerance of enteral feedings. These diseases include intestinal failure caused by necrotizing enterocolitis, short bowel syndrome, intestinal atresias, and other gastrointestinal malformations [2]–[6]. Historically, a significant proportion (up to 90%) of PN-infused infants with intestinal failure has developed cholestatic liver injury (PN-Associated Liver Injury, or PNALI), which can rapidly progress to cirrhosis [4], [6]–[15]. For this reason, PNALI is the major indication for multi-visceral transplantation (intestinal-liver) in children [4], [6]–[16]. The etiology and pathogenesis of PNALI remain largely unexplained and various treatment and prevention modalities have not been successful [17]–[19].

An intriguing property of the pathophysiology of PNALI is that its severity and chronicity are increased in those PN-dependent infants with underlying intestinal inflammation or injury, and increased intestinal permeability [20]. The importance of these alterations in intestinal physiology is demonstrated by several observations. First, the lack of enteral feedings in PN-infused infants significantly reduces intestinal motility, which favors bacterial overgrowth and further aggravates underlying inflammation [13], [21], [22]. In addition, the surgical removal of large parts of intestine may result in maladaptive peristalsis and dilation of the remaining intestine, as well as loss of the barrier function of the ileo-cecal valve, also promoting bacterial overgrowth and migration of colonic bacteria into the small intestine [20], [23]. Indeed, noninfectious chronic inflammation of the intestine is a common finding in PN-infused infants with short bowel syndrome [23]. These observations, together with the variable phenotypic expression of PNALI in infants with principally similar intestinal functional capacity and comparable amounts of PN, led us to hypothesize that differences in the composition of the intestinal microbiota might play a significant role in the pathogenesis of PNALI.

In order to study the pathogenesis of PNALI we developed a novel PNALI mouse model [24] that reproduces important features of the pathophysiology in human PN-dependent infants, including: a) combination of intestinal injury and increased permeability with infusion of PN (without enteral feeds); b) hepatocyte injury (elevated serum AST and ALT) and cholestasis (elevated serum bilirubin and bile acids) dependent on PN infusion in the presence of intestinal inflammation and increased permeability; c) liver injury associated with pro-inflammatory activation of Kupffer cells (KC) proliferation and the consequent inflammation are a characteristic of the liver histopathology in human infants and adults with PNALI; and d) both PNALI and KC activation were dependent on the presence of intestinal microbiota, a functional toll-like receptor (TLR) 4 signaling pathway, and the infusion of plant sterols, specifically stigmasterol [24]–[32].

In the present study we used this mouse model to conduct a culture-independent analysis of the composition of the intestinal microbiota in order to test the hypothesis that specific alterations in the composition of the intestinal microbiota are associated with the lipid composition of the PN solution and with the presence of KC-dependent liver injury.

## Results

### Specific alterations in the microbiome are associated with PNALI

We first compared the colon luminal microbiome of mice with both PN infusion (using soy oil emulsion-based PN solutions) and DSS-induced intestinal inflammation (PN/DSS mice, 100% of which developed PNALI and hepatic KC activation; Table 1) to the luminal microbiome of mice which were chow-fed mice, infused normal saline through a central venous catheter and had DSS induced intestinal inflammation (NS/DSS control mice, 0% of which developed PNALI). Other mouse groups included those receiving PN only, chow fed-controls, and those receiving DSS only. DNAs were purified from colonic fecal samples, PCR was conducted using barcoded primers (27F/338R) that target the V1–2 region of the bacterial small subunit ribosomal RNA (rRNA) gene [33], and products were sequenced. The resulting sequence collections constituted a taxonomic inventory of colonic bacteria for each of the animals. Approximately 1,000 (range 388–3,065) rRNA gene sequences were determined for each animal.

**Table 1:** Summary of Treatment Groups.

Treatment Group	N	DSS	NS	PN	Chow	KC*	PNALI	S24-7	Erysip.	AST**	ALT**	Bilirubin**	Bile acids**
PN/DSS (Intralipid)	14	Y	Y	Y	N	Y	**Y**	50.7%	**18.9%**	294.2 (28.6)	119 (13.6)	0.9231 (0.16)	28.42 (5.3)
NS/DSS	14	Y	Y	N	Y	N	N	49.0%	4.3%	83.64 (6.1)	27.82 (6.4)	0.2818 (0.04)	3.441 (0.7)
chow	6	N	N	N	Y	N	N	48.7%	0.1%	84.2 (7.1)	27.8 (4.3)	0.16 (0.03)	3.454 (0.4)
Chow/DSS	6	Y	N	N	Y	N	N	54.3%	0.5%	126.7 (16.9)	43.17 (4.2)	0.4167 (0.1)	7.123 (1.8)
PN (Intralipid)	8	N	N	Y	N	ND	N	44.6%	10.8%	158.7 (8.8)	65 (10.2)	0.39 (0.08)	7.84 (2.0)
PN/DSS/Abx (Intralipid)	7	Y	Y	Y	N	N	N	3.8%	6.4%	166.7 (19.6)	90 (20.5)	0.1875 (0.05)	4.86 (1.0)
PN/DSS (N-Lipids)	5	Y	Y	Y	N	N	N	58.2%	12.6%	145.3 (14.2)	53.56 (7.8)	0.3 (0.04)	6.088 (1.0)
PN/DSS (Omegaven)	5	Y	Y	Y	N	N	N	48.8%	14.1%	69.8 (7.6)	31.4 (5.2)	0.4 (0.07)	5.804 (0.9)
PN/DSS Omegaven (Stig-low)	5	Y	Y	Y	N	Y	**Y**	69.3%	12.7%	188.8 (26.7)	45 (2.4)	0.55 (0.1)	11.05 (1.0)
PN/DSS Omegaven Stig-high)	5	Y	Y	Y	N	ND	**Y**	37.1%	8.2%	262.4 (31.22)	128.2 (25.7)	0.6111 (0.04)	13.55 (2.7)

*ND = not determined;

**(mean+/−SEM).

**Figure 1:**
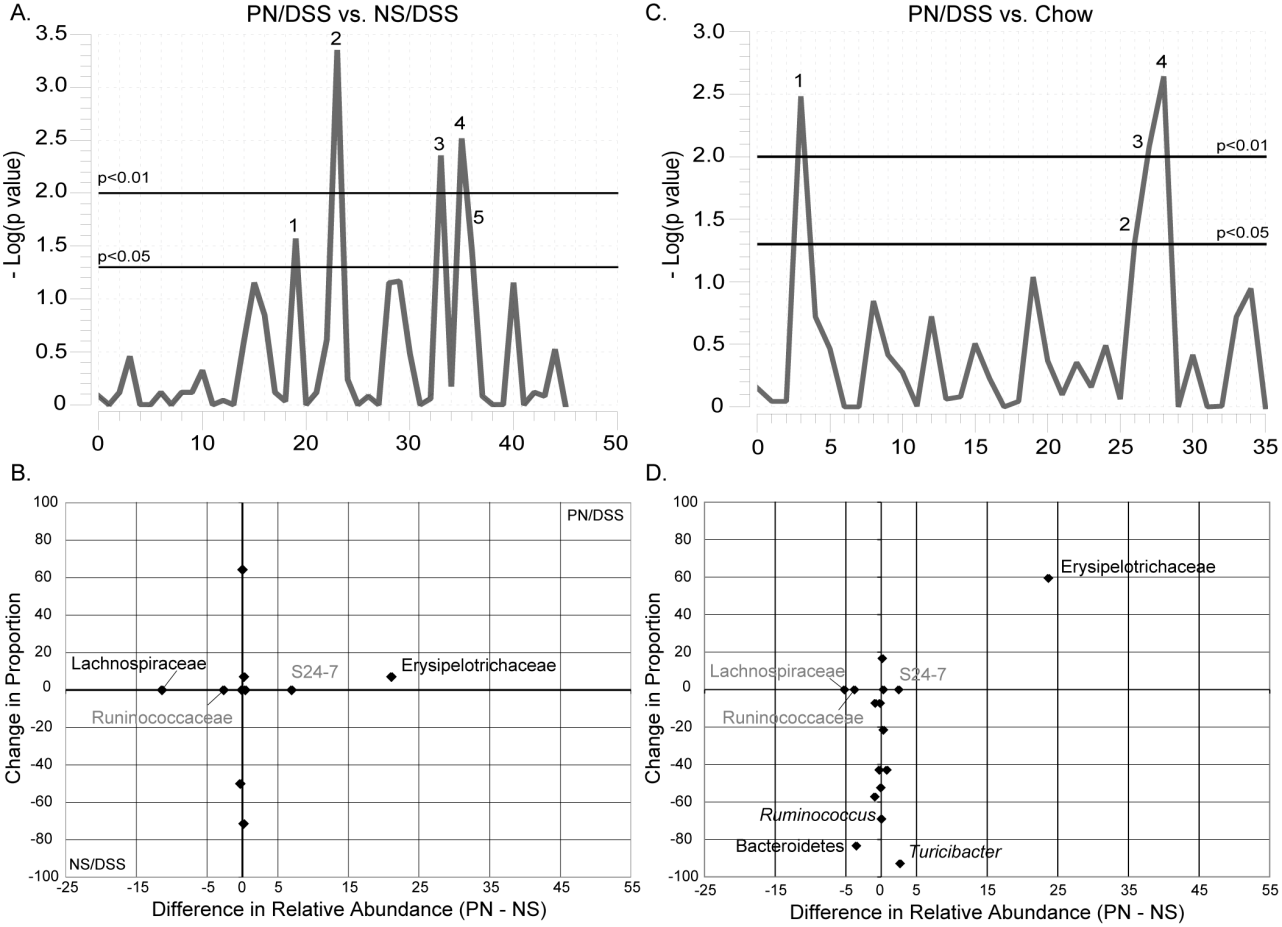
Identification of specific taxa associated with PNALI from comparison of PN/DSS and NS/DSS mice. A. Manhattan Plot showing the -log_10_ p-values from the two-part statistical test comparing 46 taxa identified between PNALI mice (PN/DSS) and controls (NS/DSS). The two-part statistic compares the proportion of samples in each group that contained a specific taxon, and the median relative abundance between groups. Taxa that were significantly different are numbered (p<0.05 and <0.01 are marked with a solid line). The numbered peaks correspond to *Clostridiales* (1), *Lachnospiraceae* (2), *Anaerotruncus* (3), *Ruminococcus* (4) and *Erysipelotrichaceae* (5). B. Change in relative abundance and proportion of animals positive for each taxon between groups is plotted. This analysis was limited to taxa present in ≥50% of individuals in either group. Taxa labels are given for groups present in >1% relative abundance. Names are shaded to correspond with statistical significant taxa (black) versus those that did not achieve statistical significance (grey). The purpose of this plot was to assess the biological relevance of each taxon identified irrespective of statistical significance. The quadrants that are labeled show where both relative abundance and proportion are higher for the group indicated. C. Manhattan plot of two-part analysis for comparison of PN/DSS versus chow. The four peaks correspond to Bacteroidetes (1), *Ruminococcus* (2), *Erysipelotrichaceae* (3) and *Turicibacter* (4). D. Change in relative abundance and proportion between PN/DSS and chow mice as described in B.

**Figure 2:**
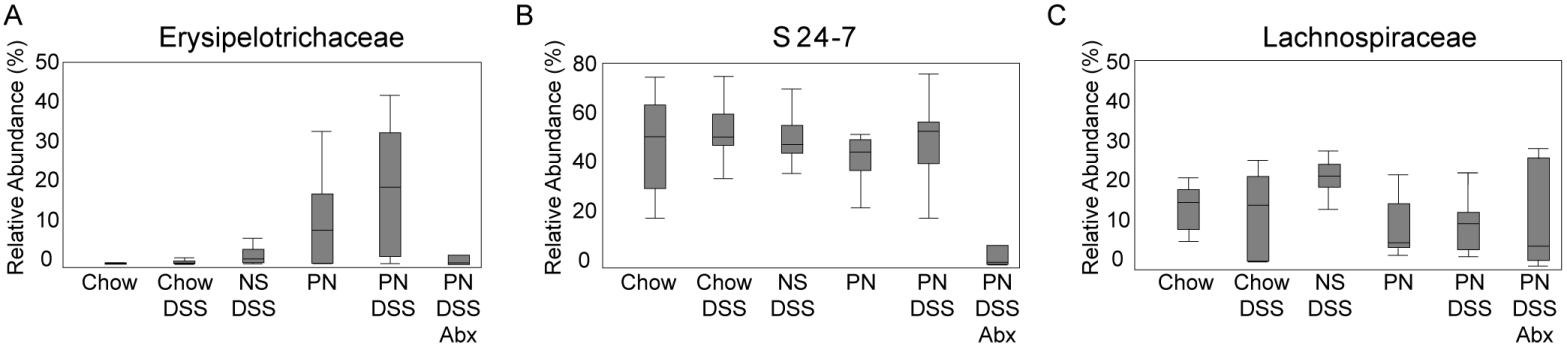
Box-whisker plots are shown comparing relative abundance across groups of mice for prominent taxa identified in two-part analyses. Statistically significant differences are marked for comparison of other groups to the PN/DSS mice (bolded; *p<0.05, **p<0.01, ***p<0.001). A. *Erysipelotrichaceae* B. S24-7 C. *Lachnospiraceae*. Box represents median and 25 and 75% ile (interquartile range, IQR) and whiskers represent 1.5x IQR. *Erysipelotrichaceae* increases in relative abundance in groups that do not have enteral feedings (PN) and those that exhibit abnormal liver function (PN/DSS). The S24-7 group is the primary Gram-negative group observed, and is eradicated by antibiotic treatment. This taxon is the most likely candidate for TLR4 activation via LPS.

We used a two-part analysis to compare the frequencies of occurrence of bacterial taxa observed in the inflamed (DSS treated) colonic microbiota with and without (saline/DSS or chow) PN, focusing on taxa that occur in ≥50% of mice in the particular groups. (Details for all taxa observed are provided tables S1 and S2 in File S2). All comparisons identified only a few statistically significant differences between the groups (Figs. 1A, C). Figures 1B and 1D display the taxon differences in terms of relative abundances in the microbiota and the proportion of occurrence. Particularly noteworthy is the dramatic bloom of *Erysipelotrichaceae* in PN only mice and more so in PN/DSS mice (the mice with PNALI). This taxon increases from a minor component, <1% of gut microbiota in chow-fed control animals, to 20–30% of the microbiota in mice with PNALI (Fig. 2A). Other significantly abundant taxa identified in the two-part analysis included some enrichment of the S24-7 group, an uncultured lineage of Bacteroidetes, and depletion of some groups upon PN, for instance *Lachnospiraceae* (Figs. 1B, D). However, as seen in the abundance measurements of these organisms (Figs. 2B, C), there are no striking differences in the control (chow) and experimental animals unless treated with antibiotics, so the association of these microbes with disease is unlikely. Detailed information for all taxa within each additional group of mice is given in Figures S1–S4.

**Figure 3:**
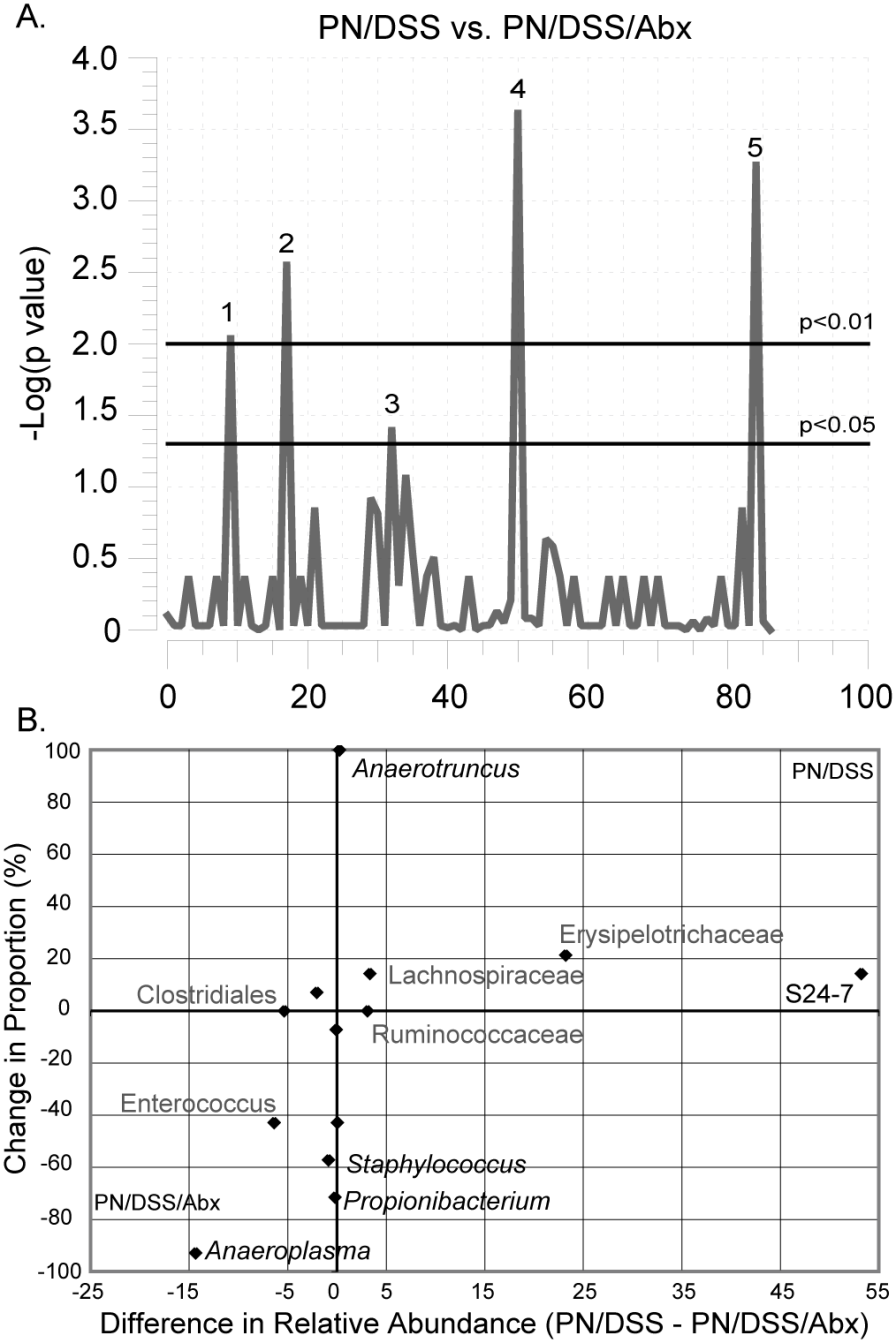
Effect of antibiotic (Abx) treatment on the mouse fecal microbiome in PN/DSS mice with PNALI. A. Manhattan plot of two part analysis of PN/DSS versus PN/DSS/Abx mice. Peaks correspond to *Propionibacterium* (1), S24-7 (2), *Staphylococcus* (3), *Anaerotruncus* (4) and *Anaeroplasma* (5). The effect of antibiotics was to depress several of the more prominent groups allowing detection of a large number of rarer taxa. B. Change in relative abundance and proportion between PN/DSS and PN/DSS/Abx mice as described in Figure 1B. Several taxa exhibited large changes in relative abundance without achieving significance (*Lachnospiraceae*, *Ruminococcaceae*, *Erysipelotrichaceae*, *Clostridiales* and *Enterococcus*).

Based on prior work using the PNALI model (PN/DSS mice), bacterial involvement was expected [24]. Analysis of the microbiota associated with PN/DSS mice compared to PN/DSS mice treated with antibiotics, which attenuates liver injury, identified taxa that were differentially present in these two groups (Figure 3A and 3B). Antibiotic treatment suppressed both *Erysipelotrichaceae* and the S24-7 lineages (Figure 3, 2A and 2B). The random forest analysis also identified Erysipelotrichaceae as an informative group for differentiation of the mice with liver injury from controls (Figure S5). These data support the importance of *Erysipelotrichaceae* in PNALI, but also highlight a potential role for the S24-7 taxon. S24-7 is expected to have a Gram-negative cellular organization that is typically associated with activation of TLR4 signaling pathways. Summary of the microbiota present in each group is provided in Figure S6.

Several additional groups of mice were examined to control for experimental manipulations required for liver injury. These included chow fed mice treated with DSS (Chow/DSS), mice infused with normal saline and allowed enteral feeds treated with DSS (NS/DSS), and mice infused with parenteral nutrition without DSS treatment (PN), none of which developed PNALI. Details of the microbiota present in these animals are given in File S1 and Tables S3–S7 in File S2. In general, there were few differences observed in comparisons between the control groups, and these were consistent with taxa identified in the primary comparisons presented (File S1, Results). Together, these data implicate the involvement of specific bacterial lineages, *Erysipelotrichaceae* and S24-7, with PNALI in our mouse model.

### Effect of soy lipids on intestinal microbiota

**Figure 4:**
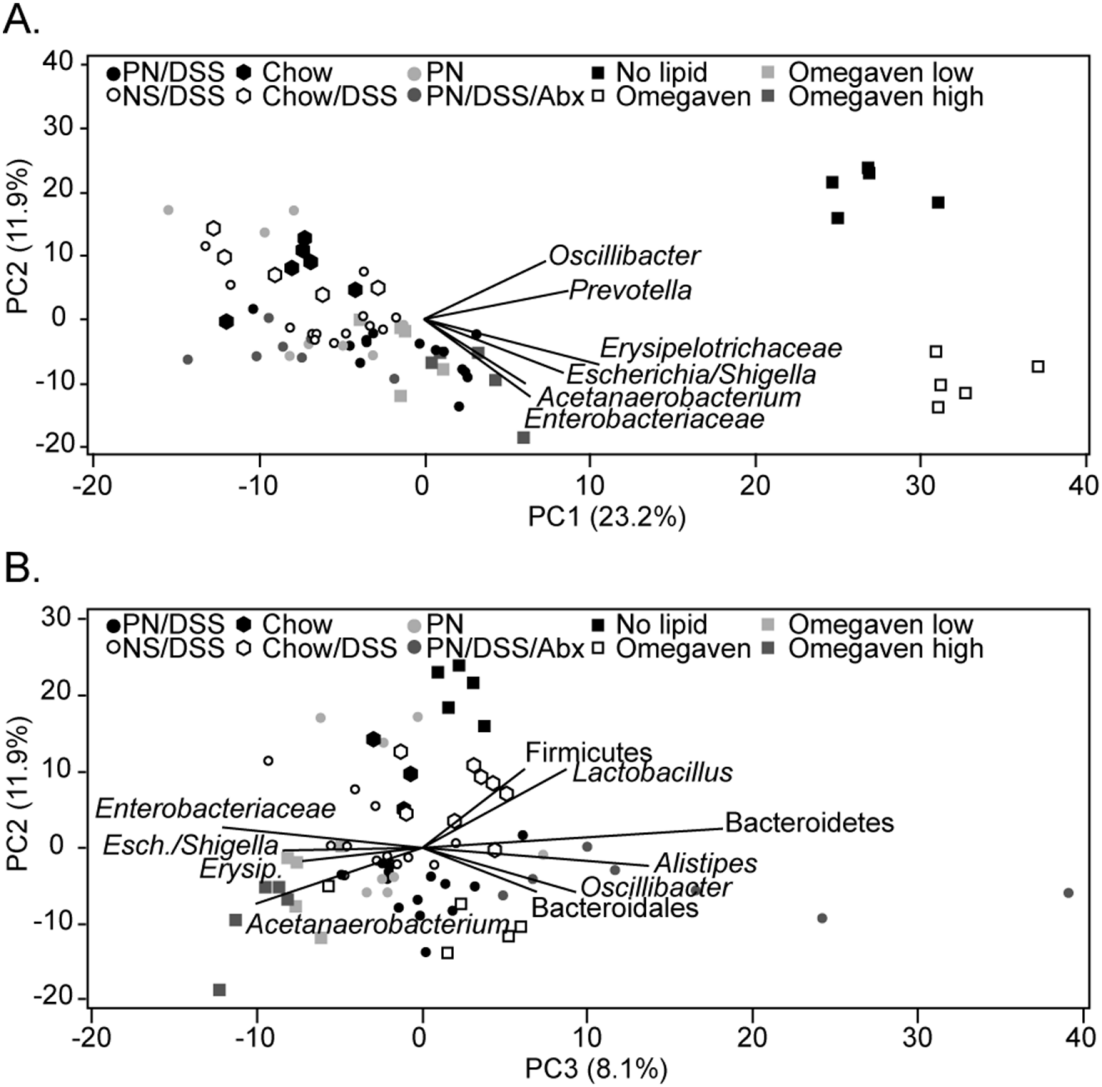
Principal component analysis of all groups of mice. A. Principal component (PC) 1 versus PC2. There is a strong signal separating groups exposed to plant material (chow or soy-based PN) from the Omegaven and no lipid control groups along PC1. This includes the two groups of animals given the stigmasterol spiked Omegaven PN formula. The taxa most influential in PC1 and PC2 are shown as vectors of the PC loadings. B. PC3 versus PC2 is shown to better distinguish between the groups exposed to plant based nutrition. Important taxa are shown as PC loading vectors as in panel A, Esch. = Escherichia and Erysip. = *Erysipelotrichaceae.*

We have previously reported that soy lipid-derived components, specifically phytosterols, within PN solutions play a key role in promoting PNALI in mice [26]. To further examine the role of PN components on the intestinal microbiota composition, four additional groups of mice with DSS-induced intestinal injury were examined and infused with modified PN solutions (Table 1): (i) PN in which the lipid fraction did not contain phytosterols but rather a fish oil-derived lipid emulsion (Omegaven, Fresenius), (ii) Omegaven spiked with a low concentration of one phytosterol, stigmasterol (1 mg/100 ml) or (iii) Omegaven spiked with a high concentration of stigmasterol (3 mg/100 ml) and (iv) a PN solution with no lipid, as previously described in detail [26]. The PN-Omegaven and PN-no lipid infused mice (which did not develop PNALI or KC activation [26]) exhibited a significantly different fecal microbiota from the other groups of mice exposed to either plant-based enteral nutrition (chow) or soy oil-based PN (which developed PNALI and KC activation [26]). Addition of stigmasterol at either concentration to the Omegaven promoted the return of the bacterial communities to resemble those detected in the original PN/DSS mice with PNALI (that were infused with phytosterol containing PN) (Figure 4). Thus, soy-derived lipid components, specifically the phytosterol stigmasterol, appear to promote selective enrichment of particular types of colonic bacteria in mice that are associated with the development of PNALI.

Because bacterial overgrowth of the small intestine and bacterial translocation are well-recognized phenomena in end-stage liver disease, including human infants with PNALI, we examined if bacteria translocate to the liver in the mouse PNALI model. Analysis of 16S rRNA gene PCR from liver tissue DNA failed to detect evidence of bacterial translocation to the liver (data not shown), suggesting that absorption/translocation of microbe associated molecular patterns (MAMPs) rather than whole bacteria drive this liver injury.

## Discussion

This is the first study to provide a molecular sequence-based analysis of alterations in the intestinal microbiota in a mouse model of PNALI. Our study provides evidence that PN-dependence and lack of enteral feeds together with specific lipid components of PN, the phytosterols, are the important drivers of microbiome alterations in mice with PNALI. Specifically, we identified a limited number of taxa that were associated with PNALI in mice. Among those, members of the *Erysipelotrichaceae* were identified as potential candidates to play a causal role in the pathogenesis of PNALI based on the correlation with liver injury and importantly, based on the association with soy-base/stigmasterol containing PN solutions. Representatives of the Gram-negative S24-7 lineage of Bacteroidetes were numerically dominant, and thus also have a potential role in PNALI. However, the S24-7 lineage did not correlate as closely with liver injury as did the *Erysipelotrichaceae*.

The direct examination of the role of phytosterols identified the importance of PN components in driving colonic bacterial community structure and liver injury (Figure 4). The Omegaven treated animals developed communities that were different from those receiving the standard soy oil-based PN solution, and did not demonstrate liver injury or KC activation. However, addition of stigmasterol, found in soy oil-based PN solutions, to the Omegaven solution was sufficient to generate communities that were similar to those in mice receiving standard PN solution and which was associated with restoration of liver injury and KC activation. These data correlate with the evidence in infants with intestinal failure that PN solutions devoid of plant sterols reverse liver injury and cholestasis [34].

Studies in both humans and mice have identified expansion of *Erysipelotrichaceae* in response to a high fat diet [35]–[37]. Investigation of cholesterol modulation through alteration of diet in a hamster model has also shown expansion of *Erysipelotrichaceae*, specifically *Allobaculum* spp., with administration of grain sorghum lipid extracts (GSL) [38]. This finding was further examined in the context of specific plant sterol administration and also identified an increase in *Allobaculum* spp. [39]. Considering the presence of plant sterols in the lipid emulsion component of PN solutions and the potential excretion of plant sterols into the gut via the biliary excretion pathway, we hypothesized that the increase in *Erysipelotrichaceae* in the PN-infused mice may be promoted by plant sterols secreted into the gut [26], [34].

We have previously shown that continuous treatment with oral broad-spectrum antibiotics during PN infusion was associated with a significant (97%) reduction of colonic luminal bacteria [24]. The current study demonstrates the dramatic reduction in antibiotic treated mice of the *Erysipelotrichaceae* and S24-7 sequences that were associated with PNALI in PN/DSS mice. These results complement our previous report that demonstrated increased absorption of LPS into the portal circulation in PN/DSS mice with PNALI [24]. Since PNALI and LPS absorption were associated with KC activation, it is noteworthy that both PNALI and KC activation were attenuated after suppression of the intestinal microbiota by treatment with broad-spectrum oral antibiotics [24]. It is important to note the *Erysipelotrichaceae* are Gram-positive organisms, and thus mechanisms in addition to TLR4 pathways that we previously identified may contribute to the pathogenesis of PNALI. Alternatively, the *Erysipelotrichaceae* could enhance intestinal absorption of microbial products from other lineages, which stimulate TLR4 through expected interactions (e.g. LPS from Gram-negative community members) [26]. Recent studies in a bile duct ligated model have demonstrated that antibiotic treatment, that suppressed intestinal microbiota to a similar degree as in our study, also led to attenuation of cholestatic liver injury. Importantly in this bile duct-ligated mouse model, antibiotic treatment did not affect intestinal permeability [40]. Based on these findings, we propose that reduction in luminal bacteria rather than restoration of intestinal barrier function is responsible for the protective effect of broad-spectrum antibiotics in the PNALI mouse model.

Our finding of a close correlation between PNALI and marked increases in abundance of specific microbes underscores that the microbiota may change early in the course of PN treatment and that intestinal dysbiosis may be an early initiating factor in the pathogenesis of PNALI. While other studies have demonstrated that the microbiome in cholestasis after bile duct ligation does not significantly change qualitatively compared to control mice [40], our study demonstrates significant dysbiosis early on in response to PN/DSS treatment. The removal of enteral feeds is likely a major contributor to the differences observed between these two cholestatic models. Our findings are consistent with those found in other mouse models in which liver injury and KC activation are also characterized by the presence of increased intestinal permeability and dysbiosis of the enteric microbiome. Importantly, in a mouse model of cholestatic liver injury, the intestinal bacterial microbiota has been shown to drive hepatic fibrosis via TLR4-dependent activation of KCs and alcohol induced liver injury was strongly reduced in *Tlr4* mutant mice [41]. Data obtained in these mouse models have also demonstrated that the pathogenesis of liver injury is associated with alterations in the intestinal microbiome (i.e. preponderance of specific bacterial taxa) and increased intestinal permeability [40]. Evaluation of *Tlr4* mutant mice (which are unresponsive to LPS) also showed attenuated PNALI, associated with attenuated KC activation [24]. While PNALI was attenuated, it was not completely prevented, by either antibiotic treatment or abrogation of TLR4 signaling, suggesting that in addition to LPS from Gram-negative bacteria, other MAMPs derived from specific bacterial lineages (e.g. TLR2 agonists from peptidoglycan derived from gram-positive bacteria) may be involved in promoting liver injury. This is consistent with microbiome examination in MyD88 deficient mice, which showed alterations in the microbiome with PN [28]. In addition, several other studies have demonstrated that the gut immune system impacts the composition of the intestinal microbiota [42]–[44] and thus there appears to exist an intricate bidirectional relationship between intestinal microbiota and immunity that may affect extra-intestinal immune signaling. We have identified the Gram-negative S24-7 lineage as well as the Gram-positive *Erysipelotrichaceae* in mice with PNALI as potential candidates to activate TLR4 and TLR2 signaling, respectively. Further evaluation of MAMPs and MAMP-sensing receptors in this PNALI model are indicated to better understand the precise signaling pathways (and potential therapeutic targets) involved in promoting this liver injury. Additional experiments with targeted antibiotic therapy to reduce specific subsets of the microbiota (e.g. *Erysipelotrichaceae* and S24-7 independently) will also help elucidate the role these prominent taxa play in PNALI.

In contrast to PN/DSS mice, PN mice did not develop PNALI, despite similarities in the composition of their intestinal microbiota. Specifically, both PN/DSS and PN mice displayed overrepresentation of *Erysipelotrichaceae*. These findings highlight that underlying intestinal injury and increased permeability are additionally required for development of PNALI in this model. Therefore, we propose that PN administration with soy oil-based emulsions and intestinal injury are necessary but not sufficient factors to promote PNALI in mice.

**Figure 5:**
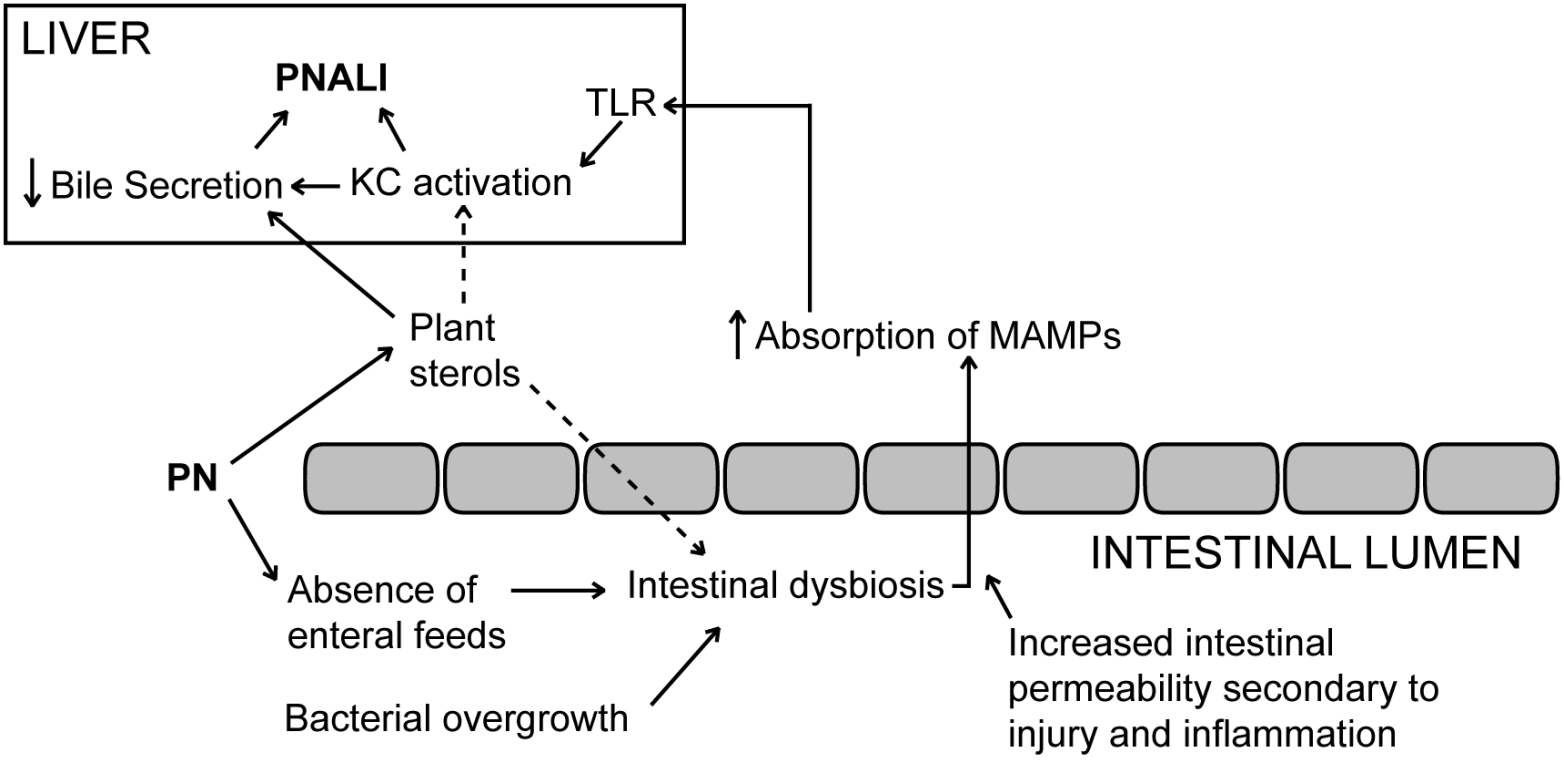
Proposed pathways involved in the pathogenesis of PNALI. Administration of PN and the associated absence of enteral feedings are the primary drivers of intestinal dysbiosis, in addition to small bowel bacterial overgrowth caused by impaired intestinal motility. In the presence of increased intestinal permeability secondary to injury and inflammation, microbe associated molecular patterns (MAMPs) derived from specific bacterial populations that thrive in the dysbiotic environment are absorbed into the portal circulation and promote TLR signaling and activation of hepatic Kupffer cells (KCs.) Soy lipid-derived plant sterols excreted into the gut via the enterohepatic circulation system in turn promote dysbiosis in addition to their effect on activation of KCs and suppression of hepatocyte bile secretion. These two latter processes culminate in PNALI and cholestasis.

Based on the findings in the present study together with our previously published data we propose that the pathogenesis of PNALI involves a complex interplay in which soy-derived plant sterols within the PN solution promote selection and overgrowth of specific bacterial species in an inflamed intestine with increased intestinal permeability, allowing MAMPs derived from these taxa to be absorbed in high quantities with subsequent activation of hepatic KCs and cytokine generation, which, combined with the cholestatic effects of infused plant sterols, trigger liver injury and cholestasis (Figure 5). Thus, early intestinal dysbiosis together with increased intestinal permeability and the infusion of plant sterols may be initiating factors that are causal to, rather than a consequence of, PNALI. Future studies using humanized gut microbiome mouse models in gnotobotic and germ free animals may yield further mechanistic proof of the role of individual microbial species of select communities in the pathogenesis of PNALI.

In conclusion, our study identifies specific changes in the microbial communities in mice with PNALI and highlights the role of PN derived plant sterols. Furthermore, the results indicate that lipid modification of PN solutions may alter specific intestinal bacterial species associated with PNALI, and thus may be a fruitful strategy for prevention or treatment of PNALI.

## Methods

### Ethics Statement

The Institutional Animal Care and Use Committee (University of Colorado, Denver) approved the research protocol [B- 82111(07)1E]. The University of Colorado, Denver is an AAALAC Accredited (#00235), Public Health Service (NIH) Assured (#A 3269-01) and USDA Licensed (#84-R-0059) Institution. All animal based research on our campus adheres to: 1) The Guide for the Care and Use of Laboratory Animals (8^th^ ed.; National Research Council; USA); 2) Public Health Service Policy on Humane Care and Use of Laboratory Animals (PHS: NIH); and 3) Animal Welfare Act (USA) and Animal Welfare Regulations (as promulgated in Title 9 Code of Federal Regulations; USA). All animals were treated humanely utilizing procedures in the approved protocol.

### PNALI Mouse model

The PNALI mouse model has recently been described [24]. Briefly, intestinal injury was produced in adult male C57BL/6 mice (8–10 weeks old) by pre-treatment with 2.5% dextran sulfate sodium (DSS) in drinking water for 4 days. DSS treatment was stopped for one day prior to assignment to a treatment regime (Table 1). Parenteral nutrition (PN) was provided through a jugular venous catheter in mice at a rate of 0.29 ml/hr providing a caloric intake of 8.4 Kcal/24 hrs. The PNALI model consisted of mice exposed to DSS followed by 7 days of PN without access to enteral feeds (PN/DSS). The administration of DSS treatment was not intended to induce a severe colitis, thus total exposure to DSS was lower than in inflammatory bowel disease mouse models. Furthermore, treatment with DSS was stopped prior to initiation of PN administration. Control mice included chow fed mice without jugular venous catheters, DSS pre-treated mice given access to chow, DSS pre-treated mice given access to chow with normal saline (NS) infused in the jugular catheter, NS infused mice that were given access to chow, and PN only mice that were not given access to chow. A group of mice received PN/DSS plus four antibiotics in the drinking water (vancomycin, streptomycin, ampicillin, and metronidazole) during the 7 days of PN [24]. Each group of mice contained between five and 14 animals. PNALI was defined by elevations in serum AST, ALT, bile acids and total bilirubin after 7 days of PN compared to control chow fed mice.

### Microbiome analysis

Fecal samples were obtained from the descending colon on the day of sacrifice (after 7 days of PN or chow diet) using sterile technique and snap frozen and stored at −70°C. Total fecal DNA was extracted as previously described [24]. DNAs were amplified in triplicate using barcoded primers (27F/338R) that target the V1–2 region of the small subunit ribosomal RNA gene [33]. A negative PCR control was run for each individual barcode. Amplicon concentration was normalized using the SequalPrep plate (Invitrogen) prior to mixing in equal amounts [45]. Sequencing was performed using the 454 titanium system (Roche) using manufacturer’s protocols. Sequence data was assigned to the appropriate sample and initial quality checks were performed using BARTAB [46]. The Omegaven and no lipid control mice were analyzed with the identical region of the rRNA gene, but with adapters compatible with the Illumina MiSeq platform. PCR was performed as described above. Pooled amplicons were quantified using the Qubit Fluorometer 2.0 (Invitrogen, Carlsbad, CA). The pool was diluted to 2 nM and denatured with 0.2 N NaOH at room temperature. The denatured DNA was diluted to 15 pM and spiked with 25% of the Illumina PhiX control DNA prior to loading the sequencer. Illumina paired-end sequencing was performed on the MiSeq platform with version 2.0 of the MiSeq Control Software, using a 500-cycle version 2 reagent kit. MiSeq paired-end sequences were sorted by sample via barcodes in the paired reads with a python script. The sorted paired reads were assembled using phrap [47]. All sequence ends (454 or MiSeq) were trimmed over a moving window of 5 nucleotides until average quality met or exceeded 20. Trimmed sequences with more than 1 ambiguity or shorter than 200 nt were discarded. Potential chimeras identified with Uchime (usearch6.0.203_i86linux32) [48] using the Schloss [49] Silva reference sequences were removed from subsequent analyses. Assembled sequences were aligned and classified with SINA (1.2.11) [50] using the 244,077 bacterial sequences in Silva 111NR [51] as reference configured to yield the Silva taxonomy. Sequences with identical taxonomic assignments were grouped to produce Operational taxonomic units (OTUs). The software package Explicet (v2.8; www.explicet.org [52]) was used for display, analysis, and figure generation of results. Sequence data is available from NCBI Sequence Read Archive under accession SRP041319.

### Statistical Analysis

Each taxon was evaluated using the Kruskal-Wallis test across all groups and used FDR correction for multiple comparisons. Random forests (RF) is a popular method employed in high dimensional datasets to identify complex data [53]. We used RF here to identify important genera for classification in a multivariate fashion. A random forest (RF) of 10,000 trees was implemented using the RandomForest R-package, all other default parameters were used. Principal component analysis was performed on the relative abundance data from each mouse. A small constant (1/total number of sequences) was added to the counts prior to the application of the centered log ratio transformation recommended for compositional data [54], [55].

Recently studies in other mouse models of liver injury have detected high levels of sequences identified only as “Bacteria” without further details, suggesting limitations with standard approaches of assessing the fecal microbiome in other mouse disease models [32], [40]. In contrast, in the present study we utilized expanded reference sequences to identify the bacteria present after standard classification approaches failed to provide identification for a significant number of sequences. This highlights the importance of the tools used to analyze microbiome data, and the need for alternative approaches when standard approaches are not successful [56]. Our expanded reference set identified a lineage of Bacteroidetes (S24-7 [57]) that was not represented in the reference taxa used for chimera detection [58]. Others have noted the importance of reference taxa selection [59].

## Supporting Information

Figure S1 **Effect of DSS pre-treatment on mouse fecal microbiomes.**
(TIF)

Figure S2 **Comparison of Chow and NS/DSS mice.**
(TIF)

Figure S3 **Effect on fecal microbiome of PN treatment vs. PN/DSS mice.**
(TIF)

Figure S4 **Effect of fecal microbiome in Chow mice versus mice who are not receiving enteral feedings.**
(TIF)

Figure S5 **Ranking of taxa identified by the random forest analysis.**
(TIF)

Figure S6 **Summary of phylum level relative abundance for primary groups of mice.**
(TIF)

File S1 **File S1 contains supplementary methods and results.**
(DOCX)

File S2 **File S2 contains supplementary tables.**
(XLSX)
