# Supplementary figures and images for: Specific Microbiome Changes in a Mouse Model of Parenteral Nutrition Associated Liver Injury and Intestinal Inflammation
Source: PLoS One. 2014 Oct 20;9(10):e110396. doi: 10.1371/journal.pone.0110396 (PMC4203793; doi:10.1371/journal.pone.0110396)

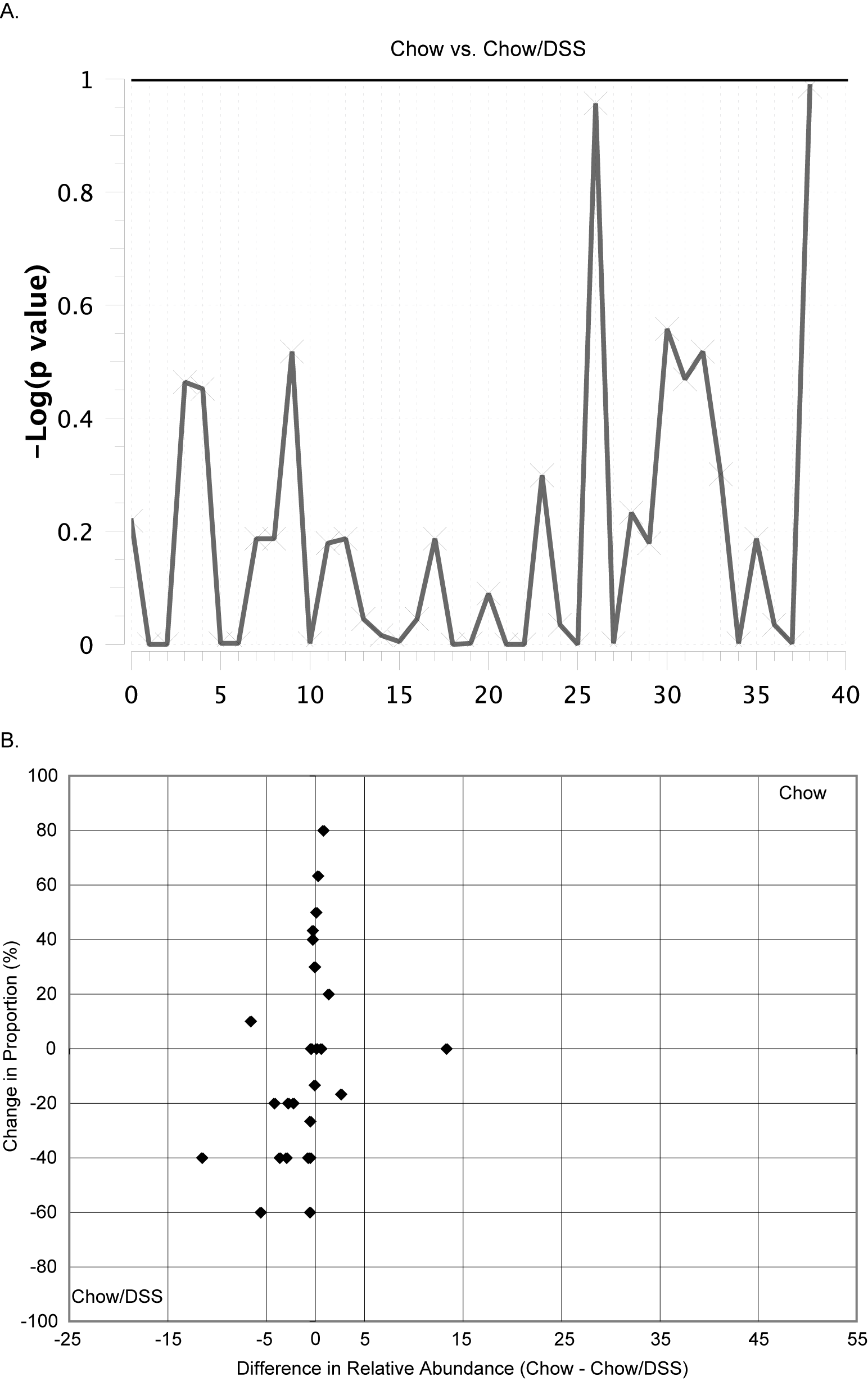

Supplement: Figure S1 — Effect of DSS pre-treatment on mouse fecal microbiomes. (TIF) [file pone.0110396.s001.tif]

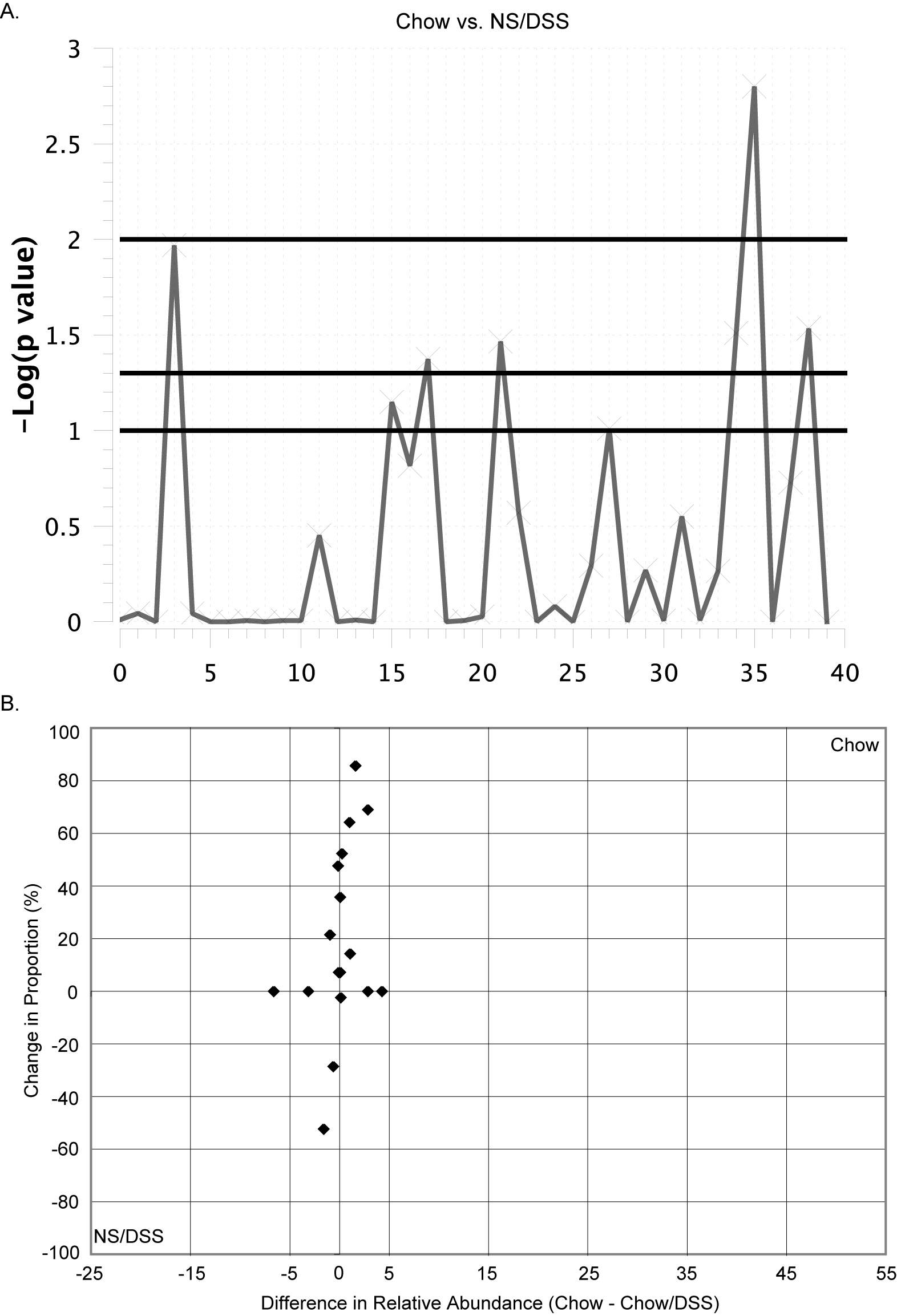

Supplement: Figure S2 — Comparison of Chow and NS/DSS mice. (TIF) [file pone.0110396.s002.tif]

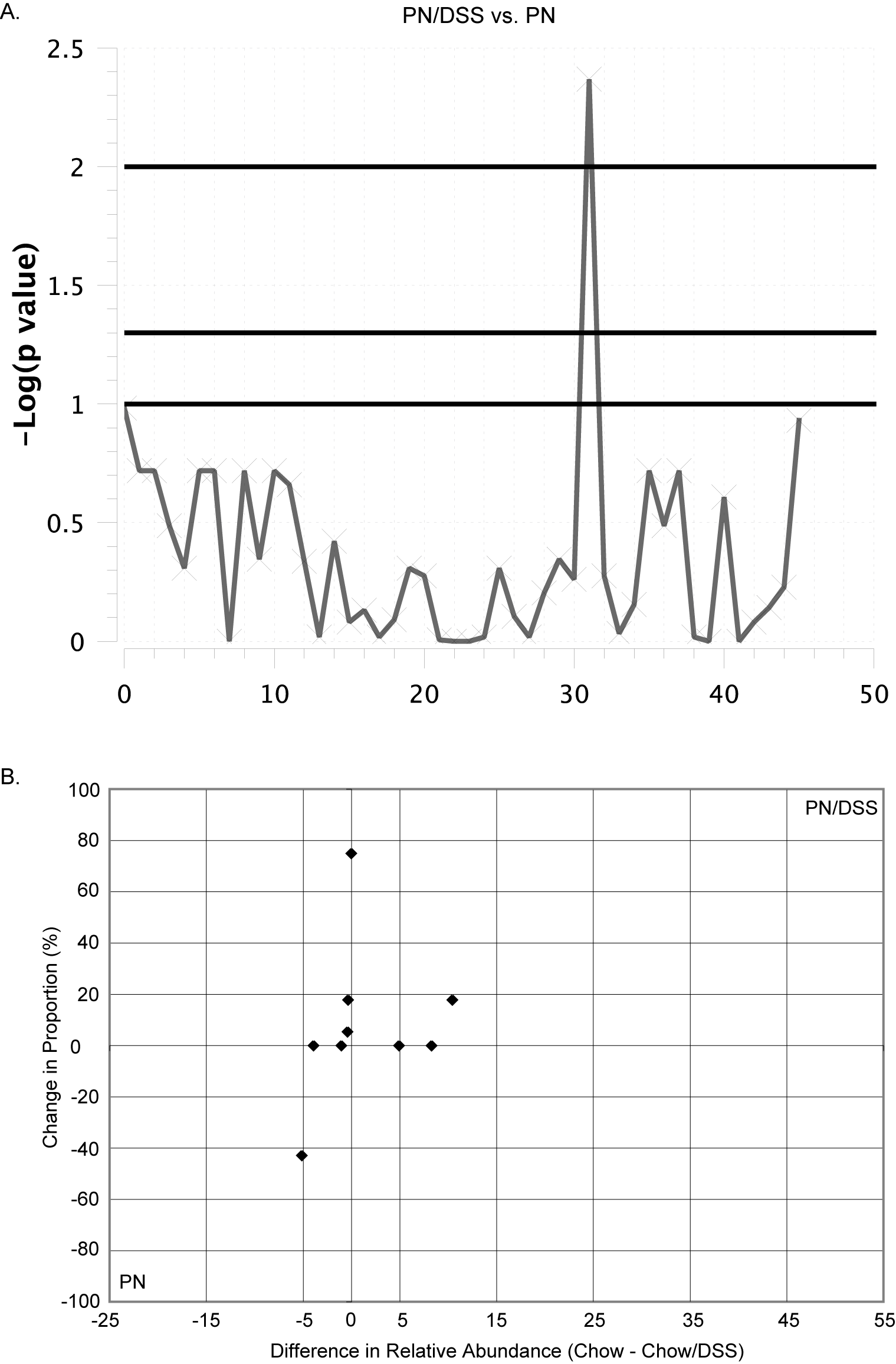

Supplement: Figure S3 — Effect on fecal microbiome of PN treatment vs. PN/DSS mice. (TIF) [file pone.0110396.s003.tif]

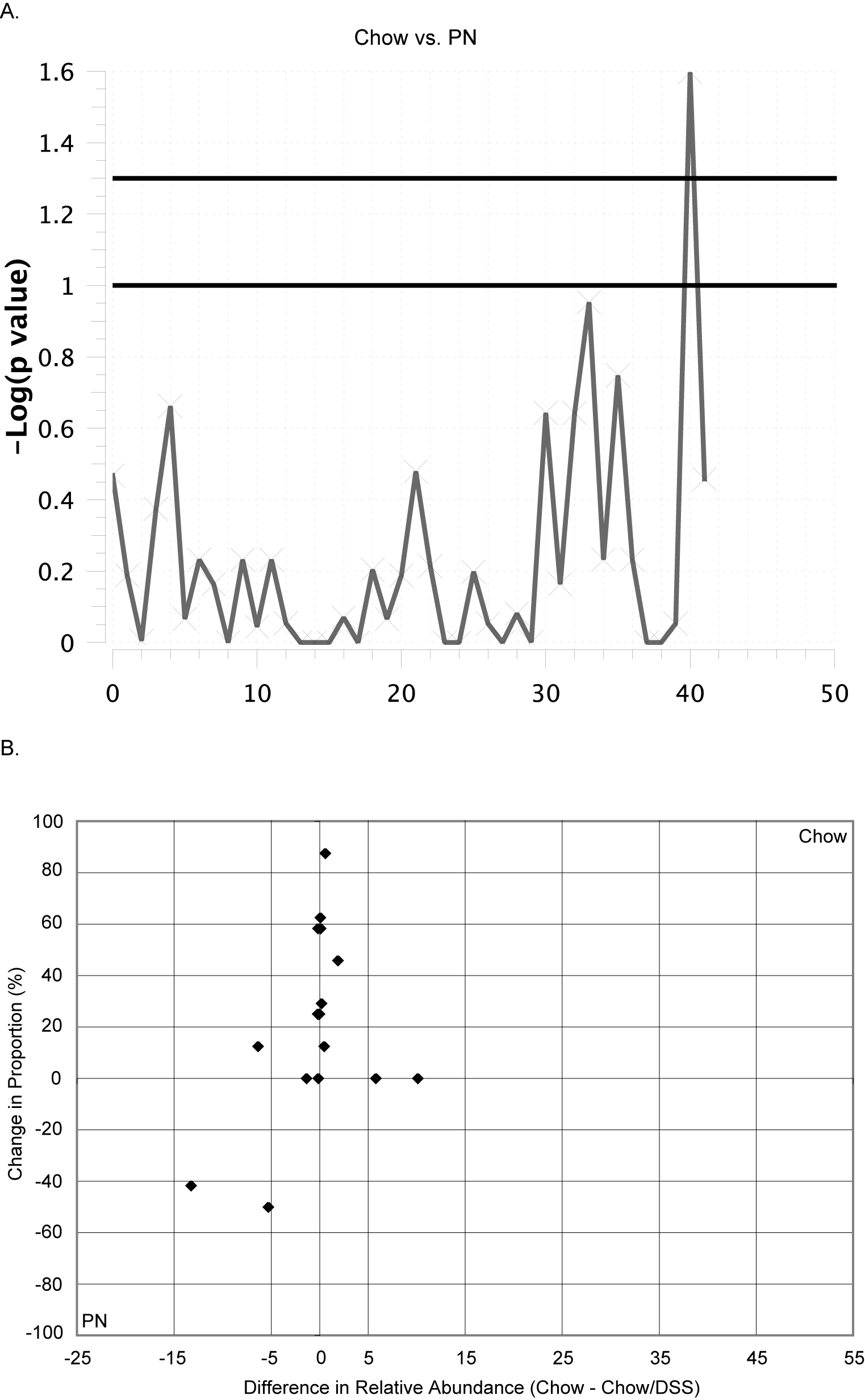

Supplement: Figure S4 — Effect of fecal microbiome in Chow mice versus mice who are not receiving enteral feedings. (TIF) [file pone.0110396.s004.tif]

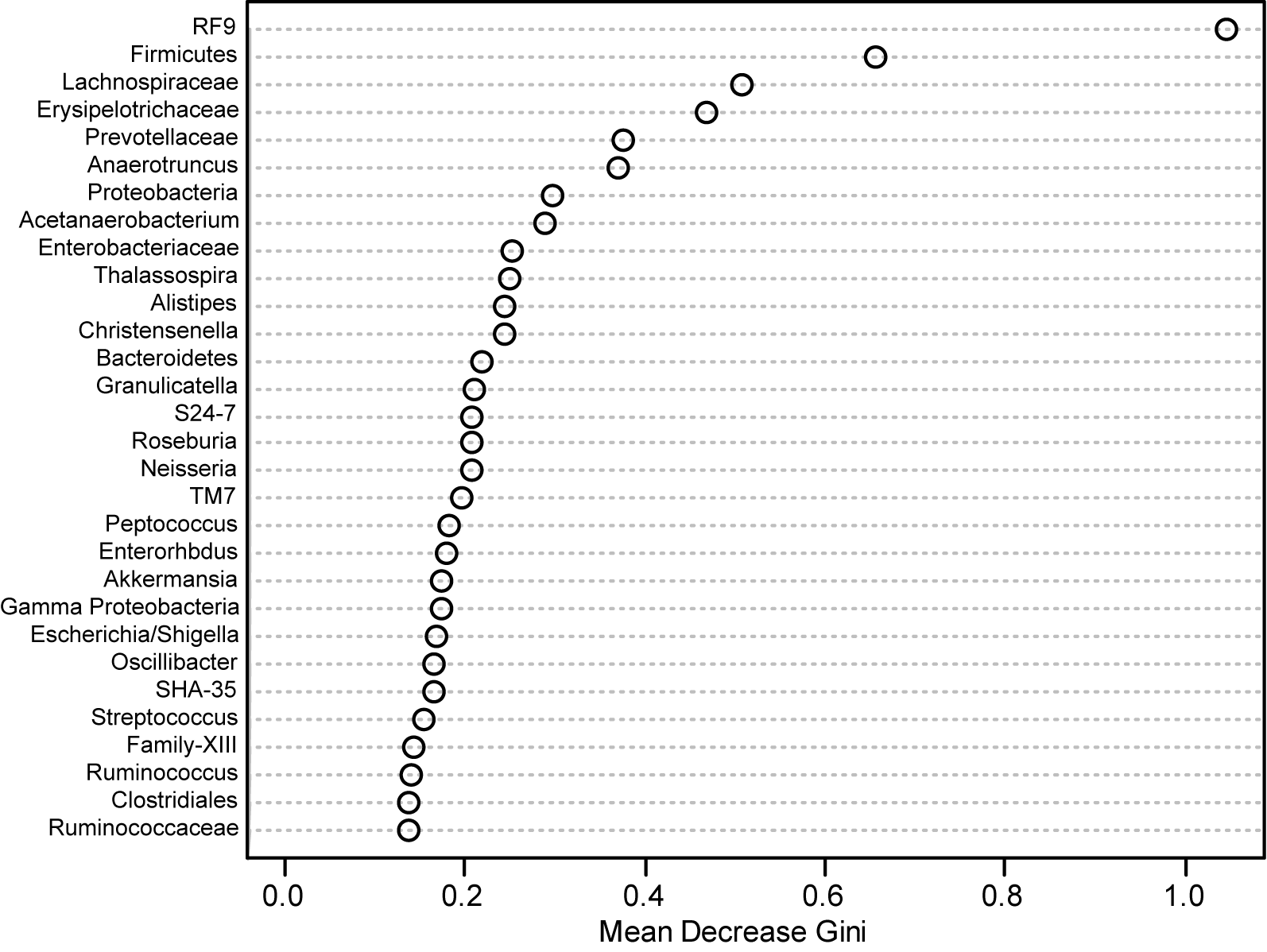

Supplement: Figure S5 — Ranking of taxa identified by the random forest analysis. (TIF) [file pone.0110396.s005.tif]

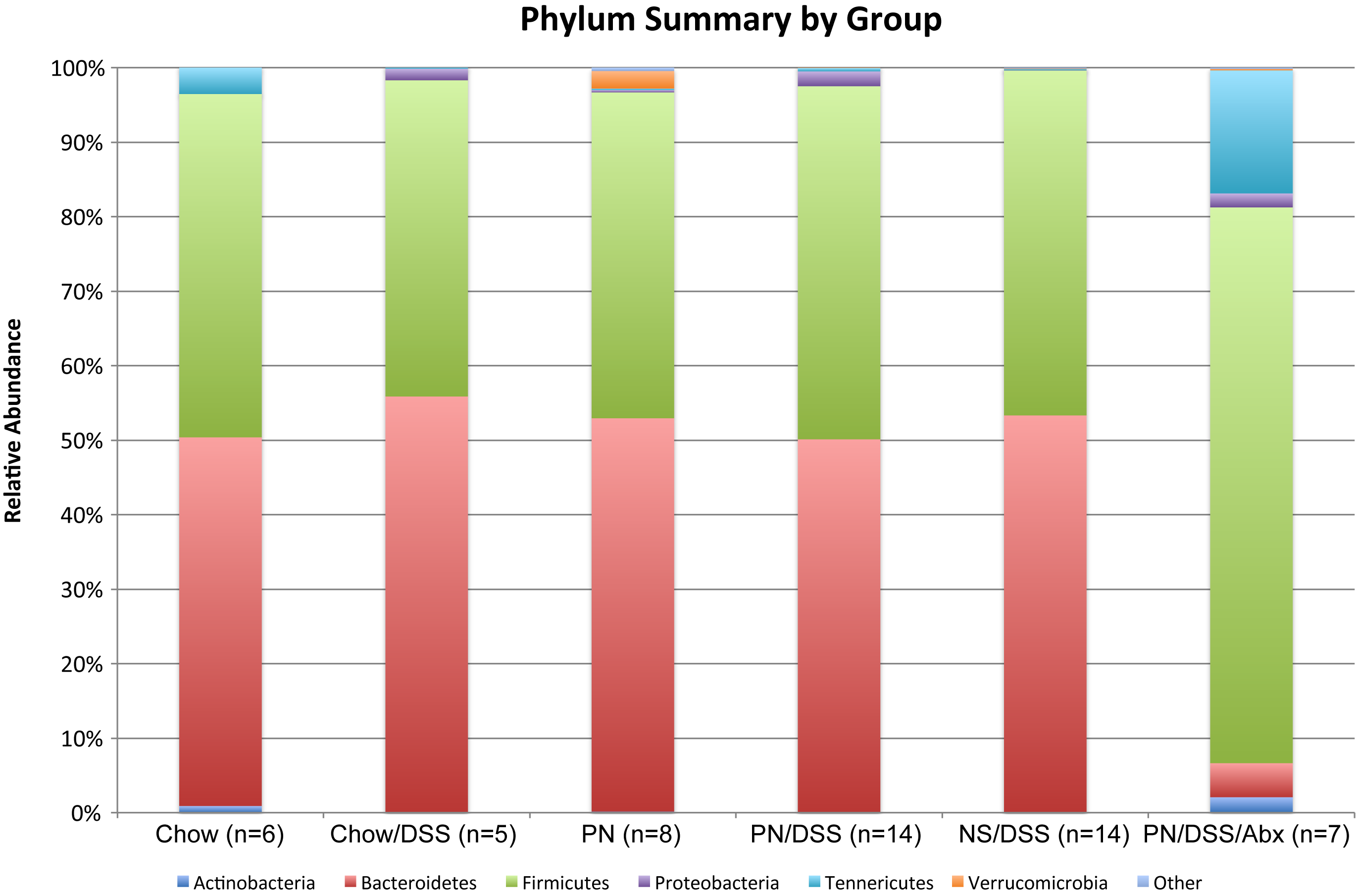

Supplement: Figure S6 — Summary of phylum level relative abundance for primary groups of mice. (TIF) [file pone.0110396.s006.tif]
