# Supplementary material for: Specific Microbiome Changes in a Mouse Model of Parenteral Nutrition Associated Liver Injury and Intestinal Inflammation
Source: PLoS One. 2014 Oct 20;9(10):e110396. doi: 10.1371/journal.pone.0110396 (PMC4203793; doi:10.1371/journal.pone.0110396)
Supplement: File S1 — contains supplementary methods and results. (DOCX) [file pone.0110396.s007.docx]

**Supplemental Methods**

**Description of PNALI Mouse Model.**

C57BL/6 wild type and syngeneic *Tlr4* mutant (B6.B10ScN-*Tlr4^lps-del^*/JthJ ([1](#_ENREF_1))) adult male mice (8-10 weeks old; Jackson Laboratories, Bar Harbor, ME) were exposed ad lib to 2.5% dextran sulphate sodium (DSS) in the drinking water for 4 days to induce intestinal injury and increased permeability ([2](#_ENREF_2)). Mice then received regular drinking water for 24 hrs (referred to as “DSS pre-treatment”) before surgical placement of a central venous catheter (CVC) (Silastic tubing, 0.012 inches internal diameter; Dow Corning, USA) into the right jugular vein followed by continuous infusion for 7 days with PN (PN/DSS) at a rate of 0.29 ml/hr providing a caloric intake of 8.4 Kcal/24 hrs. PN infused mice had access to water ad lib but not to chow during the PN infusion period. Several control groups of mice were also studied. DSS pre-treated mice were infused with NS for 7 days instead of PN (“NS/DSS”). DSS pre-treated mice that did not have a CVC placed were given free access to chow and water (DSS mice). Other mice were not DSS pre-treated but underwent CVC placement and received PN, in the same manner as PN/DSS and NS/DSS mice (“PN”). Un-manipulated control mice (“chow”) had free access to chow and water without being connected to harness-swivel system for a period of 12 days. All mice were individually housed in metabolic cages.

A group of wild type PN/DSS mice was exposed ad lib to an oral cocktail of four broad-spectrum antibiotics provided in the drinking water during the entire course of the 7-day PN infusion. The antibiotics, mixed fresh every 24 hrs, included vancomycin (1g/l), streptomycin (2g/l), ampicillin (2g/l), and metronidazole (2g/l), based on the protocol of Fagarasan et al. ([3](#_ENREF_3)).

**Supplemental Results**

**DSS-induced Intestinal inflammation promotes discrete microbiome alterations.**

The requirement for DSS-induced underlying intestinal inflammation and altered permeability to promote PNALI in this model prompted us to investigate the isolated effect of DSS treatment on alterations in the fecal microbiome. Untreated chow fed control mice were compared to chow fed DSS pre-treated animals (Chow/DSS, colonic stool was obtained from these mice on day one following completion of 4 day DSS pre-treatment) that have mild colitis and increased permeability and chow fed NS/DSS animals (colonic stool was obtained from these mice on day 8 following completion of 4 day DSS pre-treatment). In these studies we found a single environmental lineage within the Tenericutes (RF9) that was significantly different (p=0.049, Figure S1) in DSS pre-treated mice vs. untreated control mice. In contrast, we detected seven lineages to be significantly different in NS/DSS vs. chow fed controls (Figure S2). There were several other lineages that had altered relative abundance or occurrence between these two groups (Supplemental Figures S1 and S2), but with lower magnitude of change than seen in the primary comparison that identified *Erysipelotrichaceae* as significantly different (Figure 1). Information for all taxa is provided in supplemental table S4 and S5.

These results suggest that only very discrete alterations in the luminal colonic microbiome occur on day 1 after completion of DSS pre-treatment and that subsequent to DSS pre-treatment the composition of the microbiome continues to change over the next 7 days (NS/DSS mice). Furthermore, the results suggest that the differences in the microbiome detected between NS/DSS and PN/DSS mice are mainly related to absence of enteral feeds rather than intestinal inflammation in PN/DSS mice. Finally, these results suggest that over abundance of distinct taxa is specifically associated with absence of enteral feeds and development of PNALI (e.g. *Erysipelotrichaceae*).

**Absence of enteral feeds (PN) promotes specific alterations in the microbiome.**

These observations prompted us to compare PN/DSS mice (with PNALI) to PN infused mice that did not undergo DSS pre treatment (no intestinal injury and no PNALI) in order to determine the isolated effect of absence of enteral feeds on the composition of the intestinal microbiota. There were no lineages identified as significantly different between these two groups, albeit underlying intestinal inflammation in the PN/DSS mice did appear to induce some changes in relative abundance between the two groups and a shift in the proportion of animals where certain taxa were detected (Figure S3). Detailed information for this comparison is provided in Supplemental Table S6.

To investigate the effect of absence of enteral feeds on alterations in the intestinal microbiome in more detail we next compared PN mice with chow fed control mice (Figure S4). This comparison revealed two lineages that were different between Chow and PN mice, both identified in the primary analysis (Figure 1) and, intriguingly, *Erysipelotrichaceae* were associated with the absence of enteral feeds in both PN and PN/DSS mice. Details of all comparisons for all taxa are included in Supplemental Table S7.

These findings suggest that the absence of enteral feeds is the main driver in promoting specific alterations in the microbiome and demonstrate that PN specifically promotes overabundance of *Erysipelotrichaceae* while the combination of PN and intestinal injury promotes the presence of additional lineages that are associated with PNALI.

**Attenuation of PNALI after antibiotic treatment is associated with suppression of specific taxa**

We have previously reported that PNALI in mice was attenuated both in response to broad-spectrum antibiotic treatment and in mice lacking responsiveness to gram-negative LPS (Tlr4 signaling deficient mice). We therefore hypothesized that antibiotic treatment would promote specific reduction of Gram-negative bacteria (source of LPS). We investigated the composition of the luminal intestinal microbiome in PN/DSS mice in comparison to PN/DSS mice treated with antibiotics to test this hypothesis. The antibiotic cocktail did not eradicate the gut microbiota, but did reduce bacterial load in the fecal pellets by 97%. Importantly, we reported that this antibiotic treatment regimen resulted in significant attenuation of PNALI and Kupffer cell activation in these mice. Comparison of the microbiome in these animals identified eight lineages that changed significantly between the two groups (Figure 5A). The proportion of microbiota with predicted Gram-negative cell structure was greatly reduced in the antibiotic treated animals (Figure S3). Among these, the primary Bacteroidetes lineage, S24-7, was most prominently reduced in the comparison between PN/DSS and antibiotic treated PN/DSS mice. In addition, we also identified two lineages of presumed Gram-positive organisms, *Lachnospiraceae* and *Erysipelotrichaceae,* as significantly reduced in antibiotic treated PN/DSS mice. Importantly, *Erysipelotrichaceae*, which was detected with increased relative abundance in mice with PNALI was essentially eradicated in the antibiotic treated mice. Figure 6A-C shows the distribution of these three lineages across the groups based on a Kruskal-Wallis test.

Examination of taxa with altered presence between the two groups identified several other taxa that did not achieve statistical significance (Figure 5B). Table S5 provides the details on the changes between the groups.

The loss of Gram-negative lineages in PN/DSS mice treated with antibiotics supports the hypothesis that TLR4 is involved in Kupffer cell activation and liver injury in this model. However, in both *Tlr4* mutant and antibiotic treated PN/DSS mice PNALI and KC activation were significantly attenuated but not abrogated indicating that additional activation pathways may be involved in promotion of this liver injury. In keeping with this hypothesis, we also identified three prominent lineages in the comparison between PN/DSS and antibiotic treated PN/DSS mice of which is the likely trigger for TLR4 mediated activation in animals where permeability is altered. In addition, we also identified two lineages of presumed Gram-positive organisms, *Lachnospiraceae* and *Erysipelotrichaceae*, as significantly altered in the animals with liver disease and controls.

**Figure S1.** Effect of DSS pre-treatment on mouse fecal microbiomes. A. Manhattan Plot showing the output of two-part statistical test comparing 49 taxa identified between Chow and Chow/DSS mice. The two-part statistic compares the proportion of samples in each group that contained a specific taxon, and the median relative abundance between groups. No taxa were identified as significantly different. B. Change in relative abundance and proportion between Chow fed mice compared to Chow/DSS mice as described in Figure 1B for 39 taxa observed.

**Figure S2**. Comparison of Chow and NS/DSS mice. A. Manhattan Plot showing the output of two-part statistical test comparing 40 taxa identified between Chow and NS/DSS mice. The numbered peaks correspond to significantly different taxa between groups; *Bacteroidetes* (1), *Clostridiales* (2), *Lachnospiraceae* (3), *Erysipelotrichaceae* (4), *Turicibacter* (5) and RF9 (6). B. Change in relative abundance and proportion between chow mice and NS/DSS mice for 40 taxa observed. Only taxa with >1% change are identified.

**Figure S3.** Effect on fecal microbiome of PN treatment vs. PN/DSS mice. A. Manhattan plot of two-part analysis for PN versus PN/DSS comparison. A single taxon, *Anaerotruncus*, was identified with significant change between the two groups. B. Change in relative abundance and proportion between PN and PN/DSS mice as described above.

**Figure S4.**  Effect of fecal microbiome in Chow mice versus mice who are not receiving enteral feedings (PN mice). A. Manhattan plot of two-part analysis for chow versus PN comparison. A single taxon, RF9, was identified with significant change between the two groups, and is indicated on the plot. B. Change in relative abundance and proportion between Chow and PN mice as described above.

**Figure S5.** Ranking of taxa identified by the random forest analysis. Ranking of individual taxa based on the importance in distinguishing PN/DSS and NS/DSS mouse groups. The Gini-Simpson information metric is used to score the importance of each taxon based on the loss of information during classification when that taxon is excluded. The taxa identified are shown based on information content. The top two taxa identified were relatively rare, and biological importance is not clear. The third and fourth ranked taxa are *Lachnospiraceae* and *Erysipelotrichaceae*, which were identified as prominent groups within the study.

**Figure S6.** Summary of phylum level relative abundance for primary groups of mice.

1. Poltorak A*, et al.* (1998) Defective LPS signaling in C3H/HeJ and C57BL/10ScCr mice: mutations in Tlr4 gene. *Science* 282(5396):2085-2088.

2. El Kasmi KC*, et al.* (2012) Toll-like receptor 4-dependent Kupffer cell activation and liver injury in a novel mouse model of parenteral nutrition and intestinal injury. *Hepatology* 55(5):1518-1528.

3. Fagarasan S*, et al.* (2002) Critical roles of activation-induced cytidine deaminase in the homeostasis of gut flora. *Science* 298(5597):1424-1427.
